# Computational design of symmetrical eight-bladed β-propeller proteins

**DOI:** 10.1107/S205225251801480X

**Published:** 2019-01-01

**Authors:** Hiroki Noguchi, Christine Addy, David Simoncini, Staf Wouters, Bram Mylemans, Luc Van Meervelt, Thomas Schiex, Kam Y. J. Zhang, Jeremy R. H. Tame, Arnout R. D. Voet

**Affiliations:** a Laboratory of Biomolecular Modelling and Design, Department of Chemistry, KU Leuven, Celestijnenlaan 200G, 3001 Leuven, Belgium; b Graduate School of Medical Life Science, Yokohama City University, 1-7-29 Suehiro, Yokohama, Kanagawa 230-0045, Japan; c MIAT, Université de Toulouse, INRA, Castanet-Tolosan, France; d Laboratory of Biomolecular Architecture, Department of Chemistry, KU Leuven, Celestijnenlaan 200F, 3001 Leuven, Belgium; e Laboratory for Structural Bioinformatics, Center for Biosystems Dynamics Research, RIKEN, 1-7-22 Suehiro, Yokohama, Kanagawa 230-0045, Japan

**Keywords:** bioinformatics, protein structure, computational modelling, molecular simulation, structural biology, WD40 proteins, β-propeller proteins

## Abstract

β-Propeller proteins form one of the largest families of protein structures, with a pseudo-symmetrical fold made up of subdomains called blades. They are not only abundant but are also involved in a wide variety of cellular processes, often by acting as a platform for the assembly of protein complexes. WD40 proteins are a subfamily of propeller proteins with no intrinsic enzymatic activity, but their stable, modular architecture and versatile surface have allowed evolution to adapt them to many vital roles. By computationally reverse-engineering the duplication, fusion and diversification events in the evolutionary history of a WD40 protein, a perfectly symmetrical homologue called Tako8 was made. If two or four blades of Tako8 are expressed as single polypeptides, they do not self-assemble to complete the eight-bladed architecture, which may be owing to the closely spaced negative charges inside the ring. A different computational approach was employed to redesign Tako8 to create Ika8, a fourfold-symmetrical protein in which neighbouring blades carry compensating charges. Ika2 and Ika4, carrying two or four blades per subunit, respectively, were found to assemble spontaneously into a complete eight-bladed ring in solution. These artificial eight-bladed rings may find applications in bionanotechnology and as models to study the folding and evolution of WD40 proteins.

## Abstract 2

Two artificial β-propeller proteins with eight identical blades were designed, purified and crystallized. X-ray crystallography confirmed the perfectly symmetrical structures of these highly stable proteins.

## 1. Introduction   

β-Propeller proteins are widespread in nature, exhibiting a variety of functions. They consist of four to ten tandem repeats that each fold into a self-contained β-sheet with four antiparallel strands. These form the blades that are circularly arranged around a central axis, creating a propeller-like architecture (Kopec & Lupas, 2013 ▸; Pons *et al.*, 2003 ▸; Paoli, 2001 ▸). The external shape of the protein is often considered to consist of flat ‘top’ and ‘bottom’ surfaces, perpendicular to the axis of pseudo-symmetry, and a side surface between them. Each of these surfaces may take part in protein–protein interactions through a small local subset of residues, so that the roughly circular shape of the proteins makes them ideal for forming interactions with a number of different partners simultaneously. WD40 proteins are β-propeller proteins that have limited sequence conservation as a group overall, but that possess a conserved pair of tryptophan and aspartic acid residues within each blade of about 40 amino-acid residues. Genome sequencing has revealed that WD40 proteins are widespread across all kingdoms of life, and about 1% of all human structural genes are estimated to encode members of this family. A separate database has been constructed to document the roles that WD40 proteins play in processes including DNA replication, epigenetic marking of histones and ubiquitin-directed protein degradation (Schapira *et al.*, 2017 ▸).

The uniform structure of most known WD40 proteins suggests that they are an excellent starting point for the design of an artificial ring protein with perfectly conserved internal symmetry. Tandem-repeat proteins are believed to have arisen from an evolutionary process involving duplication and fusion events, creating a perfectly symmetrical intermediate protein which later diversified under evolutionary pressure (Lupas *et al.*, 2001 ▸; Orengo *et al.*, 1994 ▸; Söding & Lupas, 2003 ▸).

In recent years, several groups have created repeat proteins consisting of identical repeating domains. The majority of these consist of α-helical-based solenoid proteins such as the DARPins (Plückthun, 2015 ▸) and armadillo (Parmeggiani *et al.*, 2008 ▸), HEAT (Urvoas *et al.*, 2010 ▸), leucine-rich (Stumpp *et al.*, 2003 ▸) and tetratricopeptide-repeat proteins (Main *et al.*, 2003 ▸). The number of successfully designed globular symmetric proteins is rather smaller, but includes the pseudo-eightfold-symmetric TIM barrels that have been reconstructed out of two halves (Lang *et al.*, 2000 ▸; Höcker *et al.*, 2009 ▸), and the trefoil-repeat proteins (Broom *et al.*, 2015 ▸; Lee & Blaber, 2011 ▸). These artificial repeat proteins were built using consensus-based design or assembly from protein fragments. Recently, computational protein design has successfully been employed to create proteins with a toroid topology (Parmeggiani *et al.*, 2015 ▸; Huang *et al.*, 2016 ▸). More recently, other groups have produced symmetrical proteins, such as *de novo* designs of α-helical toroidal proteins (Doyle *et al.*, 2015 ▸).

We have previously reverse-engineered the evolutionary process of gene duplication, fusion and diversification using a computational design procedure to create Pizza6, the first perfectly symmetrical β-propeller protein (Voet *et al.*, 2014 ▸). This protein has sixfold symmetry. Tawfik and coworkers created a self-assembling β-propeller using an analysis of probable ancestral sequences but, rather than a purely computational approach, this group used libraries built from selected sequences combined with directed evolution (Smock *et al.*, 2016 ▸).

In this paper, we report the use of a modified protocol to search sequence space in the design of symmetrical proteins. β-Propeller proteins have been produced with fourfold or eightfold perfect internal symmetry, based on the crystal structure of a natural WD40 protein. The structures have been validated by X-ray crystallography and numerous biophysical methods.

## 2. Experimental methods   

### 2.1. Computational protein design   

#### 2.1.1. Computational design of Tako   

**Figure 1:**
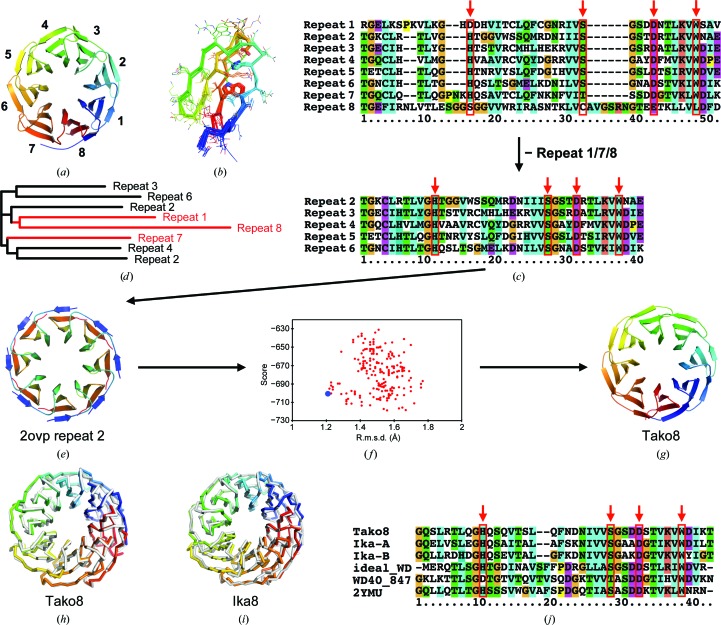
Design strategy for the Tako and Ika proteins. PDB entry 2ovp was identified as a pseudo-symmetrical protein consisting of eight repeats arranged around a central axis (*a*). The repeats were superposed (*b*). The multiple sequence alignment (*c*) and corresponding guide tree (*d*) indicate that the eighth repeat differs from the others. Each repeat from repeat 2 to repeat 6 was used individually to create perfectly symmetrical propeller backbones. The second repeat yielded the backbone with the lowest energy (*e*). The ancestral sequences, created from the sequence alignment and corresponding guide tree, were then mapped onto this backbone and the structure was optimized using *PyRosetta*. The sequence with the lowest r.m.s.d. (blue dot) from the starting backbone was chosen as the Tako8 protein (*f*). The crystallographically determined Tako8 structure (*g*) proved to be essentially identical to the design model. Overlays of the experimental models (rainbow-coloured) and designs (white) are shown for Tako8 (*h*) and Ika8 (*i*). An alignment between the repeats of Tako8, the alternating repeats of Ika and the two previous attempts at designing a perfectly symmetric WD40 protein shows the differences that may contribute to the stability of the protein (*j*). The highly conserved Trp–Ser–His–Asp motif commonly observed in WD40 proteins is shown by red arrows above the sequence alignment.

PDB entry 2ovp, an eight-bladed propeller domain of the S-phase kinase-associated protein 1A (Hao *et al.*, 2007 ▸), was selected as the starting template for the design of a symmetrical homologue (called Tako, from the Japanese for octopus; Fig. 1 ▸ *a*). The individual blades were isolated, starting at a common threonine residue, and the sequences were structurally aligned using *STRAP* (Fig. 1 ▸ *b*) (Gille *et al.*, 2014 ▸). Only the first seven blades are WD40 repeats; the eighth blade was removed from the alignment since it differs strongly in sequence and in structure. Further analysis showed that the first and seventh blades, which flank the discarded eighth blade, contain several insertions/deletions compared with the other repeats, and they were therefore also discarded (Figs. 1 ▸ *c* and 1 ▸ *d*).

The five remaining repeats were used to construct a set of consensus sequences according to the ancestral reconstruction method as previously reported. The repeat structures were also used to construct an eightfold-symmetrical backbone template by utilizing *Rosetta* symmetric docking with *C*_8_ symmetry (André *et al.*, 2007 ▸; Fig. 1 ▸ *e*). Blade 6 failed to produce templates with scores as high as the other blades, and was therefore omitted. The blades were reconnected at the conserved threonine residue to create monomeric backbones with internal eightfold symmetry. Mapping of the ancestrally reconstructed consensus sequences onto the symmetrical protein backbones (corresponding to blades 2, 3, 4 or 5) was performed using a *PyRosetta* script (Voet *et al.*, 2014 ▸, 2017 ▸), followed by energy minimization and scoring using the Talaris2014 scoring function (O’Meara *et al.*, 2015 ▸). The backbone created using the second blade gave the best results in terms of energy and backbone deviations. The sequences with the best energy scores were found to deviate significantly from the initial backbone conformation on energy minimization, so the sequence with the lowest deviation was finally chosen for experimental validation (Fig. 1 ▸ *f*). This protein was named Tako8 (Fig. 1 ▸ *g*). The eightfold identical repeat amino-acid sequence was reverse-translated into a DNA sequence with silent restriction sites for further cloning, allowing Tako2 and Tako4 to be created by simple restriction digestion and ligation.

#### 2.1.2. Computational design of Ika   

Ika8 (after the Japanese for squid) was designed using a deterministic and exact computational protein-design (CPD) tool built on top of the recent artificial intelligence prover *ToulBar*2 (Cooper *et al.*, 2010 ▸; Allouche *et al.*, 2014 ▸; Hurley *et al.*, 2016 ▸). This tool uses a fixed-backbone representation of proteins, and computes the optimal nature and orientation of side-chain rotamers for all possible amino acids. It is able to identify the global minimum energy sequence and conformation of proteins of as large as 100 residues using the energy function defined by *Rosetta* (Simoncini *et al.*, 2015 ▸). In this work, the symmetry of the structure was exploited to speed up the computation. The structure of Tako8 (PDB entry 6g6n) was divided into fourfold-symmetrical subunits of two adjacent blades. The initial template was relaxed with fourfold symmetry restraints, and the energy matrix of symmetric pairwise interactions was calculated. The CPD solver was then used to find the sequence that corresponds to the global minimum energy conformation according to the Talaris2014 energy function. Every amino-acid type was allowed at each position during the CPD calculation, except for the four residues of the conserved motif (His11, Ser27, Asp31 and Trp37), which were fixed in every blade. Additional details are given in the Supporting Information.

### 2.2. Expression and purification of recombinant proteins   

Synthetic genes were assembled (GeneArt) and inserted into pET-28b (Novagen) vector using the NdeI and XhoI sites. All plasmids were transformed into *Escherichia coli* BL21 (DE3) cells. The cells were grown at 310 K in LB medium (containing 30 µg ml^−1^ kanamycin) to an OD_600_ of 0.6. Protein expression was induced by adding isopropyl β-d-1-thio­galactopyranoside (IPTG) to a final concentration of 0.5 m*M* and shaking overnight at 293 K. The pellet was suspended in 50 m*M* phosphate pH 8.0, 300 m*M* NaCl, 10 m*M* imidazole (buffer *A*) and lysed by sonication. The lysate was centrifuged at 43 100*g* for 20 min. The supernatant was loaded onto nickel-affinity resin (5 ml HisTrap FF Crude column, GE Healthcare) equilibrated with buffer *A* before washing with 50 m*M* phosphate pH 8.0, 300 m*M* NaCl, 20 m*M* imidazole (buffer *B*). The protein was eluted with 50 m*M* phosphate pH 8.0, 300 m*M* NaCl, 300 m*M* imidazole pH 8.0 (buffer *C*) and the His tag was removed by digestion with thrombin during dialysis against buffer *A*. The protein was reloaded onto the affinity column, and the flowthrough was collected and then concentrated before loading it onto a HiLoad 16/600 Superdex 200 gel-filtration column (GE Healthcare) equilibrated with 20 m*M* HEPES pH 8.0, 200 m*M* NaCl. Selected fractions were dialyzed against 20 m*M* HEPES pH 8.0, 100 m*M* NaCl (Tako8) or 20 m*M* HEPES pH 8.0 (Ika8/4/2). The purified proteins were concentrated to 10 mg ml^−1^ using a Vivaspin 15R (Sartorius) and shown to be 95% pure by SDS–PAGE. Analysis by size-exclusion chromatography (SEC) was performed using a Superdex 200 Increase 10/300 GL column (GE Healthcare) with 20 m*M* HEPES pH 8.0 and 200 m*M* NaCl as buffer. The amino-acid sequence details of all of the proteins are given in Supplementary Table S1.

### 2.3. Crystallization, structure determination and refinement   

Protein samples were diluted to 10 mg ml^−1^ with 20 m*M* HEPES pH 8.0, 100 m*M* NaCl (Tako8) or 20 m*M* HEPES pH 8.0 (Ika2/4/8). They were subjected to crystal screening in sitting-drop 96-well plates using sparse-matrix kits (Qiagen) at 293 K. A variety of conditions gave well shaped crystals overnight, which were further optimized by hand using hanging-drop vapour diffusion. The crystallization reservoir solutions are described in Supplementary Table S2. All crystals were cryocooled using 12.5–20%(*w*/*v*) glycerol or PEG 4000 as a cryoprotectant. X-ray diffraction data were collected on beamline I04 at Diamond Light Source, Oxfordshire, England and on beamline NW12A at the Photon Factory AR, Tsukuba, Japan using PILATUS 6M-F or ADSC Quantum 270 detectors. Diffraction images were processed with *XDS* (Kabsch, 2010*a* ▸,*b* ▸) and scaling was performed with *AIMLESS* (Evans & Murshudov, 2013 ▸). Molecular replacement using the computational structures and *Phaser* (McCoy *et al.*, 2007 ▸) gave good initial models. Refinement was performed with *phenix.refine* (Adams *et al.*, 2010 ▸) and *Coot* (Emsley *et al.*, 2010 ▸). The completed structures were validated with *MolProbity* (Chen *et al.*, 2010 ▸). Data-collection and refinement statistics are given in Supplementary Table S3. The coordinates and structure-factor data have been deposited in the Protein Data Bank as entries 6g6m (Tako8 crystal No. 1), 6g6n (Tako8 crystal No. 2), 6g6o (Ika8 crystal No. 1), 6g6p (Ika8 crystal No. 2) and 6g6q (Ika4). Figures were generated in *PyMOL* (Schrödinger). Secondary structures were assigned with *DSSP* and electronic potentials were calculated using *APBS*–*PDB*2*PQR* (Touw *et al.*, 2015 ▸; Kabsch & Sander, 1983 ▸; Jurrus *et al.*, 2018 ▸).

### 2.4. Analytical ultracentrifugation (AUC)   

Sedimentation-velocity experiments were carried out with an Optima XL-I analytical ultracentrifuge (Beckman-Coulter, Fullerton, California, USA) using an An-50 Ti rotor. For sedimentation-velocity experiments, cells with a standard Epon two-channel centrepiece and sapphire windows were used. 400 µl protein solution (1.0 mg ml^−1^) and 420 µl reference buffer (20 m*M* HEPES pH 8, 100 m*M* NaCl) were used in each experiment. The rotor temperature was equilibrated at 293 K in the vacuum chamber for 2 h prior to starting each measurement. Absorbance (280 nm) scans were collected at 10 min intervals during sedimentation at 40 000 rev min^−1^. The resulting scans were analyzed using the continuous-distribution *c*(*s*) analysis module in *SEDFIT* (Schuck *et al.*, 2002 ▸). Sedimentation-coefficient increments of 200 were used in the appropriate range for each sample. The frictional coefficient was allowed to float during fitting. Partial specific volumes of the proteins, solvent density and solvent viscosity were calculated using *SEDNTERP* (Lebowitz *et al.*, 2002 ▸).

### 2.5. CD spectroscopy   

Experiments were performed on a JASCO J-1500 instrument. For CD measurements at 20°C, the proteins were diluted to 0.1 mg ml^−1^ in 20 m*M* phosphate buffer pH 7.6 using a 1 mm path quartz cuvette. Thermal unfolding experiments were carried out by heating the samples (0.25 mg ml^−1^) in a 2 mm quartz cuvette from 0 to 95°C in steps of 0.2°C. The monitored wavelength was 233 nm (Tako8) or 213 nm (Ika8/4/2).

### 2.6. Differential scanning fluorimetry   

Differential scanning fluorimetry (DSF) was performed using a QuantStudio 3 (Thermo Scientific) to determine the stability of Tako and Ika proteins. 20 µl samples (1.0 mg ml^−1^ in 20 m*M* phosphate buffer pH 7.6) were mixed with Protein Thermal Shift Dye (Thermo Scientific) and incubated from 25 to 99°C with a temperature gradient of 0.9°C min^−1^. To determine the effects of pH and salt on Tako8, buffers at different pH values were used with various concentrations of NaCl: 50 m*M* citrate pH 4, sodium acetate pH 5, MES pH 6, MOPS pH 7, HEPES pH 8 and Bicine pH 9. The fluorescence was monitored using standard excitation/emission wavelengths and the *T*_m_ of the protein was determined using the manufacturer’s software.

### 2.7. Tryptophan fluorescence spectroscopy   

Tryptophan fluorescence measurements were performed in 96-well microplates (Greiner) on a Safire2 reader (Tecan). The proteins were diluted to 0.5 mg ml^−1^ in 20 m*M* HEPES pH 8.0, 100 m*M* NaCl. To obtain tryptophan fluorescence in the unfolded state, the proteins were diluted to an equivalent concentration in 6 *M* guanidium hydrochloride (GdnHCl) and incubated for 2 h at room temperature (20°C). Excitation was performed at 280 nm and the emission spectrum was recorded between 300 and 400 nm.

### 2.8. Isothermal equilibrium denaturation (IED)   

Denaturation by GdnHCl was monitored by UV fluorescence using a Safire2 microplate reader (Tecan). GdnHCl was added in steps of 0.2 *M* to protein samples (at an OD_280_ of 0.3) in 20 m*M* phosphate buffer pH 7.6. The temperature was held at 25°C. An excitation wavelength of 280 nm was used, and emission was measured at 330 and 350 nm. All data were analysed using a two-state model (folded/unfolded; Scholtz *et al.*, 2009 ▸). Six parameters were fitted to the data: Gibbs free-energy difference (Δ*G* ^o^), sensitivity towards denaturation (*m*), and the intercepts (γ^0^_F_ and γ^0^_U_) and the slopes (*m*_F_ and *m*_U_) of the folded (F) and unfolded (U) transition baselines. The change in the free energy of folding for different proteins, ΔΔ*G*, was calculated according to ΔΔ*G* = (*C*_*m*,*A*_ − *C*_*m*,*B*_)(*m*_*A*_ + *m*_*B*_)/2 (Pace & Scholtz, 1997 ▸). All thermodynamic parameters are shown in Supplementary Table S4.

## 3. Results and discussion   

### 3.1. Protein structures   

**Figure 2:**
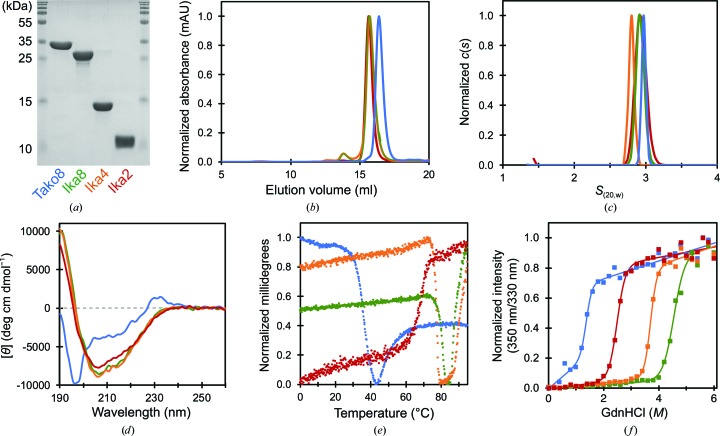
Purification and characterization of Tako8 and Ika proteins. The SDS–PAGE confirmed the purity of the proteins (*a*). In all figures, Tako8, Ika8, Ika4 and Ika2 are represented by blue, green, orange and red lines, respectively. The analytical SEC (*b*) and AUC (*c*) show that the proteins are monodisperse, with the expected mass. The UV CD spectra indicate β-structure (*d*) which can be used to observe thermal denaturation (*e*). The resistance of the protein towards denaturation by GdnHCl was monitored using tryptophan fluorescence. Fractional denaturation was determined from the ratio of emission at 350 and 330 nm after excitation at 280 nm (*f*). Both temperature and chemical denaturation indicate that the Ika proteins are more stable than Tako8 and that the Ika proteins increase in stability with polypeptide length. All proteins were dissolved in 20 m*M* phosphate buffer pH 7.6 (no NaCl) for folding and stability experiments (*d*, *e*, *f*).

**Figure 3:**
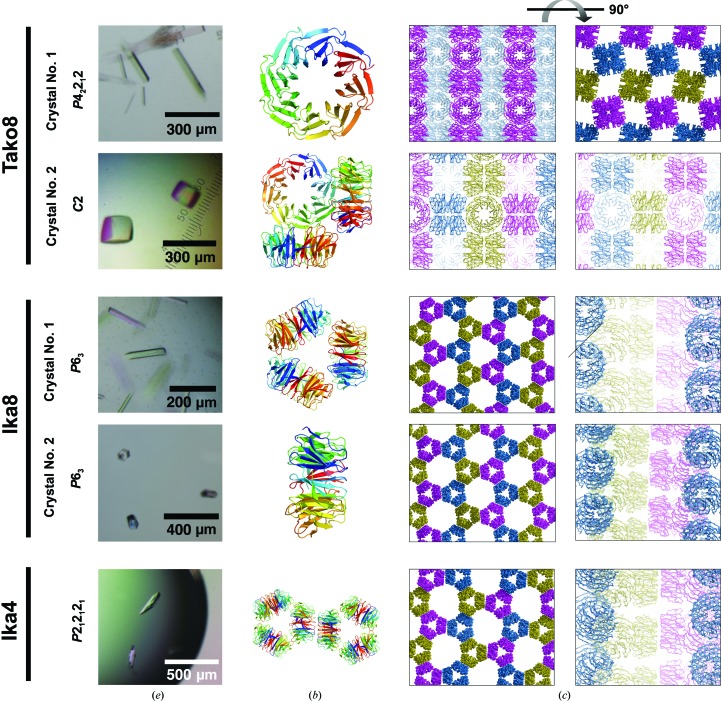
Crystals and crystal packing of the Tako and Ika proteins. (*a*) Pictures of the crystals grown under different conditions. (*b*) Protein structures in the asymmetric unit, with each chain coloured blue to red from the N-terminus to the C-terminus. (*c*) Two orthogonal views of the crystal packing. The tetragonal Tako8 crystals form a rhomboid cavity of about 40 Å across. The Ika8 and Ika4 crystals show large open channels of about 90 Å across with a honeycomb-like arrangement.

The Tako8 protein expressed at a very high level, was soluble and could readily be purified and concentrated in the presence of 200 m*M* sodium chloride (Fig. 2 ▸ *a*). Both SEC and AUC indicated a monodisperse monomeric species with the expected molecular weight (Figs. 2 ▸ *b* and 2 ▸ *c*). The protein crystallized under a variety of conditions, but crystals in space group *P*4_2_2_1_2, with a single monomer in the asymmetric unit, gave the highest resolution diffraction to 1.7 Å resolution (Tako8 crystal No. 1; Fig. 3 ▸). A second crystal form belonging to space group *C*2, with three molecules in the asymmetric unit, diffracted to 2.0 Å resolution (Tako8 crystal No. 2; Fig. 3 ▸). The two final models showed an overall C^α^ r.m.s.d. of 0.3 Å from each other and of 1.0 Å from the designed structure (Fig. 1 ▸ *h*). There is one Ramachandran outlier residue in each blade: Gly9 and its equivalents. The entire protein is well represented in the electron-density map (Supplementary Fig. S1). The eightfold symmetry of the protein is reflected in the water structure within the central cavity, which is 11 Å across. The tetragonal crystals show large solvent channels, and the monomeric form shows a pseudo-cubic arrangement of molecules around large symmetrical cavities. In both crystal forms the Tako8 molecules show a conserved interaction at the top side where two proteins meet face to face. The crystal-packing interaction is mediated by a hydrogen-bonding pattern with eightfold symmetry. The difference between the two crystal forms arises from different water-mediated interactions at the outer rim of the toroid (Supplementary Fig. S2*a*).

**Figure 4:**
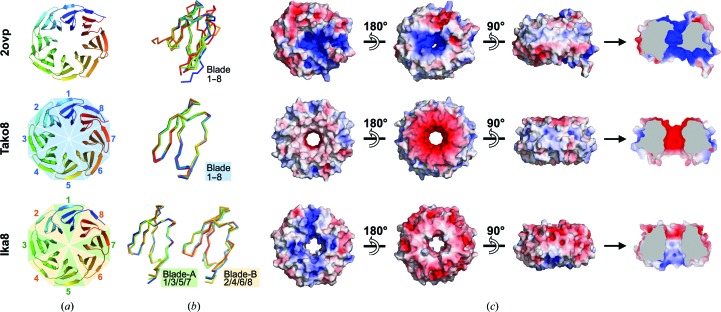
Comparison of the parent, Tako and Ika propeller structures. (*a*) The overall β-propeller domain is depicted as a cartoon coloured from blue to red from the N-terminus to the C-terminus. (*b*) Superposition of the individual blades of the different proteins indicates that the parent protein does not exhibit a conserved backbone geometry, while the eight blades of Tako8 can be perfectly aligned. In the case of Ika8 only the alternating *a* and *b* repeats can be perfectly aligned. The differences in symmetry are also clear from the electrostatic potential as calculated by *APBS* (Touw *et al.*, 2015 ▸; Kabsch & Sander, 1983 ▸; Jurrus *et al.*, 2018 ▸) (*c*). In the case of Tako8 the interior of the toroid is clearly highly negatively charged, while for Ika8 the charges are more evenly distributed.

Tako8 was found to have highest stability around pH 8, but to be extremely unstable in the absence of salt. Increasing the salt concentration led to increased stability over the tested pH range (Supplementary Fig. S3). Dialysing the protein against buffer without salt led to rapid unfolding and loss of the protein within 24 h. To determine whether polypeptides carrying a different number of Tako blades could be expressed, clones were created carrying two, four, seven or nine blades. Tako2 produced inclusion bodies, while Tako4 and Tako7 gave no apparent expression. Tako9 could be expressed and crystallized in identical conditions to Tako8; it forms an eight-bladed structure with a single unfolded blade (data not shown). Each Tako blade carries a highly negative charge at the entrance to each side of the central cavity, formed by Asp21, Asp29 and Asp30 and their equivalents (Figs. 4 ▸ *c*). In the absence of salt, these negatively charged rings of carboxyl groups, spaced at roughly 9 and 6 Å, respectively, may well prevent the stable assembly of subunits carrying fewer than eight blades. Lowering the pH to counter the negative charges of the Asp residues did not improve the stability, probably owing to protonation of the histidine, which is part of the highly conserved Trp–Ser–His–Asp bridge. The concentration of negatively charged residues reflects their high conservation in the parental template structures, so Ika8 was designed as a fourfold-symmetrical protein, allowing the search algorithm to identify compensating interactions between adjacent blades.

In contrast to Tako, Ika expressed well as a polypeptide with two, four or eight blades. Each form could be purified (Fig. 2 ▸ *a*). SEC and AUC revealed that each Ika construct exists as a single eight-bladed propeller in solution, indicating that oligomerization can occur (Figs. 2 ▸ *b* and 2 ▸ *c*). Moreover, all of the Ika proteins showed an identical secondary structure (Fig. 2 ▸ *d*). Ika8 produced two crystal forms in space group *P*6_3_, with one or three copies in the asymmetric unit. Ika4, however, crystallized in space group *P*2_1_2_1_2_1_ (Fig. 3 ▸). The crystals of Ika8 and Ika4 are built from three separate eight-bladed molecules with their symmetry axes perpendicular to a common threefold axis (Fig. 3 ▸). In one crystal form of Ika8 this axis is crystallographic, but in the other (and in the Ika4 crystals) it is noncrystallographic. The C^α^ r.m.s.d. between the designed and experimentally observed Ika8 structures was 1.7 Å, and the r.m.s.d. between experimental models was roughly 0.5 Å (Fig. 1 ▸ *i*). In all crystal forms, the Ika proteins assemble as trimers which stack on top of each other into pillars. Unlike in the Tako8 protein, the conserved inter­actions stabilizing the crystal assembly involve hydrogen bonds between the outer rims of neighbouring molecules, while the interactions at the top face are mediated by water molecules and are not conserved (Supplementary Fig. S2*b*). Ika2 was not found to crystallize, possibly because of higher flexibility. The extra N-terminal residues may also interfere with crystal contacts, suggesting that the local packing common to the Ika4 and Ika8 crystals is strongly preferred over other possibilities (Fig. 3 ▸ *c*, Supplementary Fig S2*c*).

### 3.2. Comparing the Tako and Ika proteins   

The melting/denaturation curves show that the Ika proteins are more stable than Tako8, especially under low-salt conditions. Thermal stability measurements by CD, which reflect the loss of secondary structure upon protein unfolding, demonstrated that the *T*_m_ value of Ika8 is roughly 50°C higher than that of Tako8 (Fig. 2 ▸ *e*, Supplementary Table S4). A similar result was also observed using DSF, which assesses tertiary integrity (Supplementary Fig. S4, Supplementary Table S4). All of the proteins except Ika2 were aggregated after heat-stress measurements (Supplementary Fig. S5). The chemical denaturation curve was determined by the addition of GdnHCl and monitoring the resulting peak shift in typtophan fluorescence. Thermodynamic parameters from the fitting curve also demonstrated the higher stability of Ika8 compared with Tako8 (Fig. 2 ▸ *f*, Supplementary Fig. S6 and Supplementary Table S4). A comparison with the template structure PDB entry 2ovp was not possible, as in this structure the β-propeller domain is preceded by a large α-helical-rich N-terminal domain, and the β-propeller domain alone failed to express.

The higher stability of Ika suggests that the increased design space offered by the lower fourfold symmetry was well exploited by global optimization using the Talaris2014 energy function. The high symmetry of the Tako protein is evident from the crystal structure, and determination of the space group proved challenging (Tako8 crystal No. 2). Indexing in space group *I*432 gave a low *R*_p.i.m._ (overall, 0.008; outer shell, 0.089) and only two blades in the asymmetric unit. This is only a quarter of the total tertiary structure, and was only made possible by the perfectly symmetrical tertiary structure of this monomeric protein. However, the final refinement was performed in space group *C*2, with a complete propeller in the asymmetric unit, as this yielded an improved *R* and *R*_free_.

The highly symmetric nature of the designer proteins is not only apparent in the crystal symmetry, as discussed above, but can also be observed at the tertiary-structural level. The individual repeats of the Tako8 protein can be perfectly aligned. In comparison, only the alternating repeats of the Ika proteins can be superposed, in contrast to the template structure where the overlap of the individual blades is much poorer. The symmetry is also visible in the electrostatic potential map, where eightfold and fourfold symmetry can be observed for Tako8 and Ika8, respectively, with the exception of small differences owing to crystal-packing inter­actions; such charge symmetry is absent from the template protein (Fig. 4 ▸).

The Tako and Ika crystals show large solvent channels and cavities, which are reflected by the high solvent content of 64–68%. Such porous assemblies may have applications in the biotemplating of nanoparticles. Recently, metal nanoparticles have been created within crystals of lysozyme (Wei *et al.*, 2011 ▸; Liang *et al.*, 2013 ▸). Our highly symmetrical frameworks may be expected to template crystalline nanoparticles more easily than an asymmetrical protein such as lysozyme. This is further demonstrated by the work of Abe and coworkers, who engineered proteins to control the porosity of their crystal assembly in order to bind certain ligands (Abe *et al.*, 2017 ▸). Furthermore, the assembly of these highly symmetric proteins could be further controlled by chemical ligands, as demonstrated previously for naturally occurring highly symmetric multimeric proteins with the help of organic ligands (Guagnini *et al.*, 2018 ▸; McGovern *et al.*, 2012 ▸; Sontz *et al.*, 2015 ▸).

### 3.3. Comparison with other proteins   

Tako and Ika are the first successfully designed eight-bladed WD40-repeat proteins for which structures have been confirmed crystallographically. WD40 proteins are typically sevenfold pseudo-symmetric, and in this case the protein template contained only seven WD40 repeats plus one other blade. This did not prevent ancestral reconstruction from producing stable perfectly symmetrical variants. Previously, Paoli and coworkers attempted a consensus-based design, which yielded protein that tended to fold as a molten globule and was prone to aggregation (Nikkhah *et al.*, 2006 ▸). Later, Baker and coworkers attempted to make a symmetrical WD40 protein using a straightforward *Rosetta*-based approach, but the resulting design proved to be unstable as a monomer and could not be validated using crystallography (Parmeggiani *et al.*, 2015 ▸). It is notable that the highly conserved Asp–His–Ser–Trp motif was not preserved in this design (Fig. 1 ▸ *j*). The loss of any key residue or residue pair within the architecture of the protein may cause a dramatic change in stability and may well account for the observed instability. Tako and Ika show that ancestral design coupled with a more exhaustive sequence search, while preserving the anchor residues, can produce stable, symmetrical proteins.

**Figure 5:**
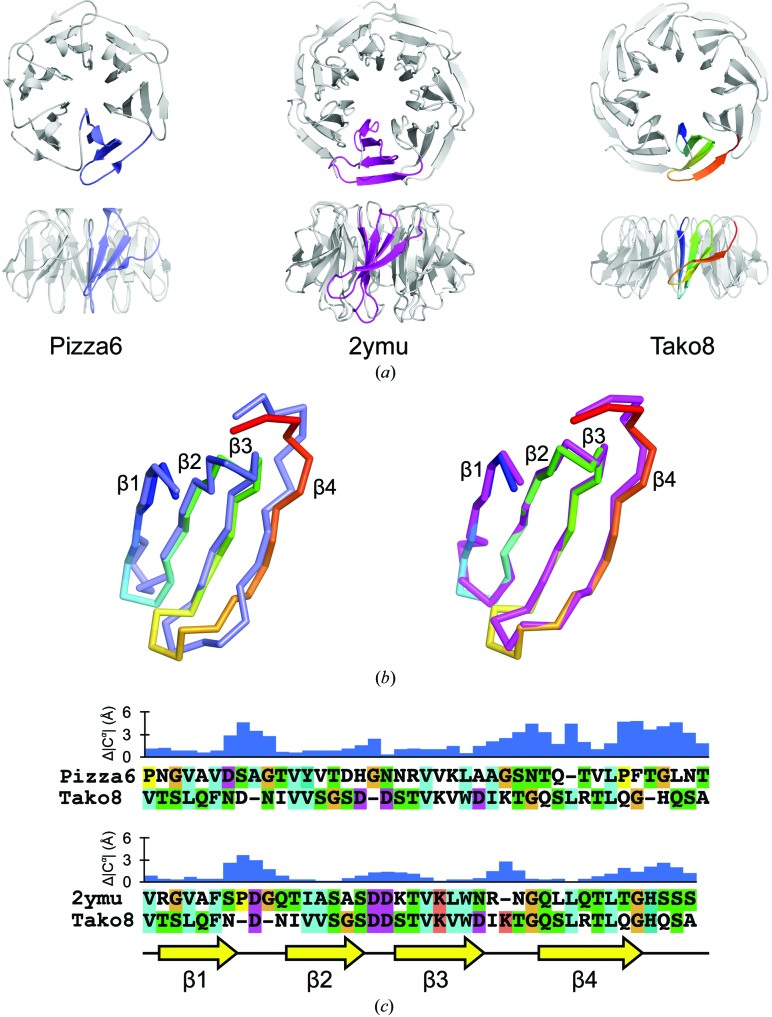
Comparison of Pizza6, PDB entry 2ymu and Tako8. Single blades of Pizza6 (PDB entry 3ww9; coloured blue), a highly symmetric naturally occurring seven-bladed propeller (PDB entry 2ymu, coloured magenta) and Tako8 (rainbow) were isolated (*a*) and superposed (*b*). The inner three strands of each blade superpose closely, while the turn between the first and the second strand and the region between the end of the fourth strand and the subsequent connecting loop deviate by up to 5 Å for Pizza6 and 3 Å for PDB entry 2ymu (*b*). Structure-based sequence alignments of a single Pizza blade *versus* Tako and of PDB entry 2ymu *versus* Tako are shown in (*c*), with the β-strands indicated as yellow arrows. The bar graphs indicate the C^α^ deviations after least-squares superposition of the two models, showing the larger differences at the C-­terminal end of the blade. The deviation between the first and second strands as well as the twist of the fourth strand is larger when the difference in number of repeats is larger. These differences influence the relative position of the blades around the central axis and help to control the number of blades in the complete propeller.

Superimposing the blades of the sixfold-symmetric designer protein Pizza6 and the eightfold designer protein Tako8 reveals that the blade architecture is almost identical (Fig. 5 ▸). This is especially true for the three inner strands of each blade, even though Pizza and Tako are derived from different β-propeller subfamilies: NHL and WD40, respectively. The outer strand and loop connecting adjacent blades are structurally unconserved and are most likely to influence the twist and relative position of each blade and help to determine the number of blades per ring. We conclude that the eight-bladed architecture is very stable since we were unable to express a seven-blade equivalent, and a nine-blade polypeptide only exhibited an eight-bladed ring. Likewise, Pizza was only stable as a sixfold-symmetric protein, and even when the number of repeats was varied the protein reassembled into larger complexes consisting of a fixed number of six-bladed units. The propeller architecture shows a strong preference for a particular symmetry, in sharp contrast to the 11-mer ring-shaped TRAP protein, which can easily adapt to 12-fold symmetry (Heddle *et al.*, 2006 ▸).

No perfectly symmetrical WD40 protein has previously been reported, but there is one WD40 structure in the PDB with a highly repetitive sequence (PDB entry 2ymu). This natural protein has an unknown function and consists of 14 tandemly repeated WD40 motifs that fold into two separate seven-bladed propeller units. Comparison of Tako8 with the 2ymu consensus sequence reveals a highly conserved backbone architecture overall, with differences in the inner bottom and outer top strands connecting the repeats, consistent with the suggestion that these regions help to control the overall symmetry. The differences between the sevenfold and eightfold symmetrical proteins are however smaller (up to 3 Å) than those between the sixfold and eightfold symmetrical proteins, which can deviate by up to 5 Å (see Fig. 5 ▸). These deviations arise from both insertions and deletions as well as different amino-acid compositions. Further studies will be required to understand more precisely how the edge strands influence the backbone conformations, in order to use them to tune the symmetry of propeller proteins.

Tako8 is the third protein that we have successfully designed using ancestral sequence reconstruction. Currently, there is a limited set of fully symmetrical designer proteins, which includes trefoil proteins (Lee & Blaber, 2011 ▸; Broom *et al.*, 2015 ▸), a five-bladed β-propeller-like lectin (Smock *et al.*, 2016 ▸) and the sixfold-symmetrical NHL-repeat Pizza proteins (Voet *et al.*, 2014 ▸, 2017 ▸). Owing to their symmetry, these proteins are less complex than their natural counterparts and therefore serve as models to study protein folding and evolution (Lee & Blaber, 2011 ▸; Broom *et al.*, 2015 ▸; Xia *et al.*, 2016 ▸). Tako8 should prove an ideal tool to study WD40 repeats, and since it contains a buried tryptophan residue in every repeat denaturation may be monitored by fluorescence (Fig. 2 ▸ *f*, Supplementary Figs. S6 and S7).

## 4. Conclusions   

We have successfully designed and structurally characterized a perfectly symmetrical WD40 protein with eightfold symmetry (Tako8). From this template, a derivative fourfold-symmetrical protein called Ika8 was created. The latter was designed using a novel computational procedure and is able to reassemble from individual repeating units. Both proteins are stable and can be purified in high yields, but the altered charge distribution in Ika8 allows it to remain folded under low-salt conditions. It may well be possible to create other similar proteins that withstand low ionic strength, while maintaining the eightfold symmetry, by giving greater weight to the charge distribution throughout the design procedure. X-ray crystallo­graphy confirms that the expected Tako8 and Ika8 structures agree closely with the experimental models. Applications have yet to be demonstrated for artificial symmetrical proteins, although the designer lectin Mitsuba has been shown to bind Raji cancer cells selectively (Terada *et al.*, 2017 ▸), and we have shown that a Pizza-derivative protein could biomineralize a 19-atom nanocrystal of cadmium chloride through metal-coordinating histidines (Voet *et al.*, 2015 ▸). Similarly, the central channel of Tako8 and Ika8 may be able to nucleate catalytic metal clusters, with potential uses in chemistry and medicine. It is hoped that these proteins will prove to be valuable building blocks for various bionanotechnological applications and in evolutionary studies of the WD40 protein family.

## Supplementary Material

PDB reference: computationally designed Tako8 protein, space group *P*4_2_2_1_2, 6g6m

PDB reference: space group *C*2, 6g6n

PDB reference: computationally designed Ika8 protein, crystal packing No. 1 in space group *P*6_3_, 6g6o

PDB reference: crystal packing No. 2 in space group *P*6_3_, 6g6p

PDB reference: computationally designed Ika4 protein, 6g6q

Supplementary Tables and Figures.. DOI: 10.1107/S205225251801480X/jt5028sup1.pdf
