# Supplementary material for: Computational design of symmetrical eight-bladed β-propeller proteins
Source: IUCrJ. 2019 Jan 1;6(Pt 1):46–55. doi: 10.1107/S205225251801480X (PMC6327176; doi:10.1107/S205225251801480X)
Supplement: Supplementary file 1 [file m-06-00046-sup1.pdf]

IUCrJ

Volume 6 (2019)

Supporting information for article:

Computational design of symmetrical eight-bladed β-propeller proteins

Hiroki Noguchi, Christine Addy, David Simoncini, Staf Wouters, Bram Mylemans, Luc Van Meervelt, Thomas Schiex, Kam Y. J. Zhang, Jeremy R. H. Tame and Arnout R. D. Voet

S1. Encoding symmetrical energy for global optimization

The computational design task was modelled using a fixed backbone representation, the Dunbrack 2010 rotamer library and the pairwise decomposable energy function Talaris2014. The symmetry definition file and the 4-fold symmetric protein subunit were generated using the Rosetta script *make_symmdef_file.pl*, using the *PSEUDO* symmetry mode. The protein backbone was relaxed 10 times using the Rosetta Relax application, with symmetry restraints and the energy function Talaris2014, and the lowest energy model was kept. The symmetric pairwise interaction energy matrix was computed starting from a 4-fold symmetric subunit, along with the symmetry definition file, Talaris2014 energy function and the Dunbrack 2010 rotamer library (the −ex1 flag was activated in order to get extra rotamers at χ_1).

In order to benefit from guaranteed energy optimization and provably identify the global minimum energy conformation, the symmetric energy matrix was extracted and converted to a Cost Function Network using in-house PyRosetta scripts, as already described in (Simoncini *et al.*, 2015). The CFN was given to the CPD version of the CFN solver Toulbar2 (available in the “cpd” branch of the git repository at http://github.com/toulbar2/toulbar2), which identified and proved the optimality of the global minimum energy conformation with the options -dee: -O=-3 -B=1 -A -s --cpd --scpbranch.

Table S1 Protein sequence, Molecular weight and Theoretical pI of Tako/Ika series

Protein	Sequence	*Molecular weight (Da)*[a]	*Theoretical pI*[a]
Tako8	MGSSHHHHHHSSGLVPRGSHM<br>**GQSLRTLQGHQSAVTSLQFNDNIVVSGSDDSTVKVWDIKT**<br>**GQSLRTLQGHQSAVTSLQFNDNIVVSGSDDSTVKVWDIKT**<br>**GQSLRTLQGHQSAVTSLQFNDNIVVSGSDDSTVKVWDIKT**<br>**GQSLRTLQGHQSAVTSLQFNDNIVVSGSDDSTVKVWDIKT**<br>**GQSLRTLQGHQSAVTSLQFNDNIVVSGSDDSTVKVWDIKT**<br>**GQSLRTLQGHQSAVTSLQFNDNIVVSGSDDSTVKVWDIKT**<br>**GQSLRTLQGHQSAVTSLQFNDNIVVSGSDDSTVKVWDIKT**<br>**GQSLRTLQGHQSAVTSLQFNDNIVVSGSDDSTVKVWDIK**<br>GS	34991.39	5.48
Ika8	MGSSHHHHHHSSGLVPRGSHM<br>**GQELVSLEGHQSAITALAFSKNIVVSGAADGTIKVWDILT**<br>**GQLLRDHDGHQSEVTALQFKDNIVVSGAKDGTVKVWYIGT**<br>**GQELVSLEGHQSAITALAFSKNIVVSGAADGTIKVWDILT**<br>**GQLLRDHDGHQSEVTALQFKDNIVVSGAKDGTVKVWYIGT**<br>**GQELVSLEGHQSAITALAFSKNIVVSGAADGTIKVWDILT**<br>**GQLLRDHDGHQSEVTALQFKDNIVVSGAKDGTVKVWYIGT**<br>**GQELVSLEGHQSAITALAFSKNIVVSGAADGTIKVWDILT**<br>**GQLLRDHDGHQSEVTALQFKDNIVVSGAKDGTVKVWYIGT**	34384.72	5.46
Ika4	MGSSHHHHHHSSGLVPRGSHM<br>**GQELVSLEGHQSAITALAFSKNIVVSGAADGTIKVWDILT**<br>**GQLLRDHDGHQSEVTALQFKDNIVVSGAKDGTVKVWYIGT**<br>**GQELVSLEGHQSAITALAFSKNIVVSGAADGTIKVWDILT**<br>**GQLLRDHDGHQSEVTALQFKDNIVVSGAKDGTVKVWYIGT**	17407.60	5.53
Ika2	MGSSHHHHHHSSGLVPRGSHM<br>**GQELVSLEGHQSAITALAFSKNIVVSGAADGTIKVWDILT**<br>**GQLLRDHDGHQSEVTALQFKDNIVVSGAKDGTVKVWYIGT**	8919.04	5.67

Expressed protein sequence (all), after purification protein sequence (underlined) and computationally designing sequence (Bold).

a: Molecular Weight (Da) and Theoretical pI value are calculated on the purified sequences by ProtParam (https://web.expasy.org/protparam/).

Table S2 Overview of crystallization conditions.

Protein	Crystal No.	Protein conc. (mg/ml)	Reservoir Solution	Cryo protectant	Crystallization time
Tako8	1	10	0.1M MES pH6.0, 1.6M $AmSO_4$	20% Glycerol	1 week
	2	10	0.1M citrate pH5.0, 3.4M NaCl	20% Glycerol	1 week
Ika8	1	10	0.1M Tris-HCl pH8.0, 0.2M $CaCl_2$, 15% Glycerol, 16% (w/v) PEG4000	20% (w/v) PEG4000	1 week
	2	10	0.1M MES pH6.0, 1.0M LiCl, 20% (w/v) PEG6000	20% Glycerol	1 week
Ika4		10	0.1M HEPES pH7.5, 0.2M sodium acetate, 25% (w/v) PEG3350	12.5% Glycerol	1 week

Table S3 Data-collection and refinement statistics of X-ray structures

Values in parentheses are for the outer shell.

	Tako8		Ika8		Ika4
	Crystal No.1	Crystal No.2	Crystal No.1	Crystal No.2	
**PDB code**	6G6M	6G6N	6G6O	6G6P	6G6Q
**Data collection**					
Diffraction source	DLS, I-04	DLS, I-04	PF-AR, NW12A	DLS, I-04	DLS, I-04
Wavelength (Å)	0.9795	0.9795	1.00000	0.9795	0.9795
Resolution range (Å)	45.20–1.70 (1.73–1.70)	46.77-2.00 (2.05-2.00)	46.62-2.05 (2.09-2.05)	48.41-2.40 (2.49-2.40)	49.07-2.50 (2.54-2.50)
Space group	$P4_22_12$	$C2$	$P6_3$	$P6_3$	$P2_12_12_1$
a, *b*, *c* (Å)	90.40, 90.40, 91.85	93.55, 132.30, 81.01	218.61, 218.61, 53.37	127.37, 127.37, 53.87	53.52, 219.19, 264.91
α, β, γ (°)	90, 90, 90	90, 125.26, 90	90, 90, 120	90, 90, 120	90, 90, 90
Reflections (measured/unique)	1,110,243/42,512	365,876/54,194	731,453/90,235	397,488/19,765	98,6217/109,112
Completeness (%)	100.0 (100.0)	99.9 (100.0)	98.0 (85.3)	100.0 (99.9)	99.7 (99.9)
Mean $I/\sigma(I)$	28.8 (2.0)	18.5 (3.6)	27.9 (5.0)	19.9 (3.3)	11.6 (2.7)
Multiplicity	26.1 (25.3)	6.8 (7.0)	8.1 (4.2)	20.1 (20.4)	9.0 (9.2)
R_{pim}‡	0.016 (0.430)	0.025 (0.304)	0.019 (0.145)	0.018 (0.245)	0.046 (0.277)
CC(1/2)§	1.000 (0.740)	0.999 (0.862)	0.999 (0.880)	0.999 (0.910)	0.997 (0.841)
Wilson B factor (Å^2)	26.1	45.4	28.3	63.0	41.6
**Refinement statistics**					
Resolution range (Å)	45.20-1.70	46.77-2.00	46.62-2.05	48.41-2.40	49.07-2.50
R factor$^{\Pi}$(%)/$R_{free}{}^{\Pi}$(%)	16.7/19.6	24.3/26.6	22.8/25.3	21.2/26.3	19.2/22.9
No. of atoms in structure					
Protein	2,449	7,257	7,202	2,403	14,272
Ligand	20	0	24	0	0
Water	227	218	218	36	431
R.m.s. deviations from ideal					
Bond lengths (Å)	0.007	0.003	0.002	0.003	0.002
Bond angles (°)	1.009	0.620	0.490	0.599	0.509
Chiral volumes (Å^3)	0.073	0.050	0.049	0.058	0.051
Ramachandran plot, residues in (%)					
Most favorable region	92.45	92.94	95.15	95.89	97.22
Additional allowed region	7.55	6.74	4.64	4.11	2.78
Average *B* factor (Å^2)	30.0	57.0	28.0	71.0	43.0

‡: $R_{pim} = \sum_{hkl} [1/(N-1)]^{1/2} \sum_i |I_i(hkl) - I(hkl)| / \sum_{hkl} \sum_i I_i(hkl)$, where $I_i(hkl)$ is the intensity of an observation, <I(hkl)> is the mean value for that reflection and the summations are over all reflections. Π: R factor = $\sum_{hkl} ||F_{obs}| - |F_{calc}|| / \sum_{hkl} |F_{obs}|$, where F_{obs} and F_{calc} are the observed and calculated structure-factor amplitudes, respectively. The free *R* factor was calculated with 5% of the data excluded from the refinement.

Table S4 Protein stability parameters

	T_m (°C)		The GdnHCl denaturing parameters			
Protein	CD	DSF	ΔG^o (kJ/mol)	*m* value (kJ mol^{-1} M^{-1})	C_m (M)	$\Delta\Delta G^a$ (kJ/mol)
Tako8	36.2	58.9	32.32 ± 11.11	23.50 ± 7.82	1.38 ± 0.66	--
Ika8	83.8	86.9	56.43 ± 10.37	12.42 ± 2.35	4.54 ± 1.20	-56.9^b
Ika4	76.6	83.7	79.46 ± 19.29	21.48 ± 5.23	3.70 ± 1.27	-52.3^b / 14.3^c
Ika2	69.4	80.6	40.39 ± 6.41	16.24 ± 2.55	2.49 ± 0.56	-22.1^b / 29.5^c

a: $\Delta\Delta G = (C_{m\,A} - C_{m\,B})(m_A + m_B)/2$.

b: The reference protein “A” is Tako8.

c: The reference protein “A” is Ika8.

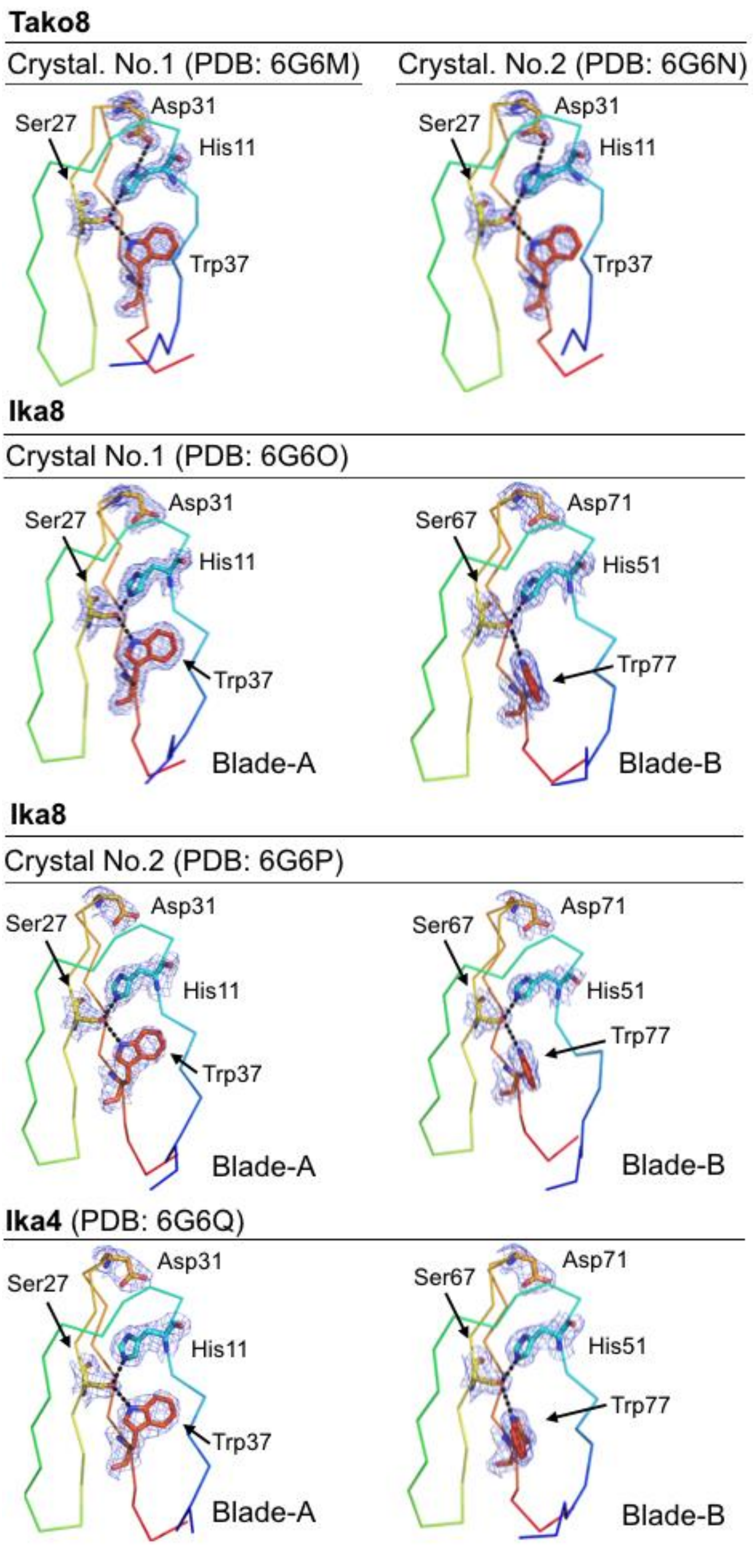

Figure S1 The 2Fo-Fc electron density maps covering the conserved Trp-Ser-His-Asp motif. The maps are contoured at a level of 1.5 σ. A single unique blade is shown for each model as a Cα ribbon, coloured from blue to red, N to C terminus.

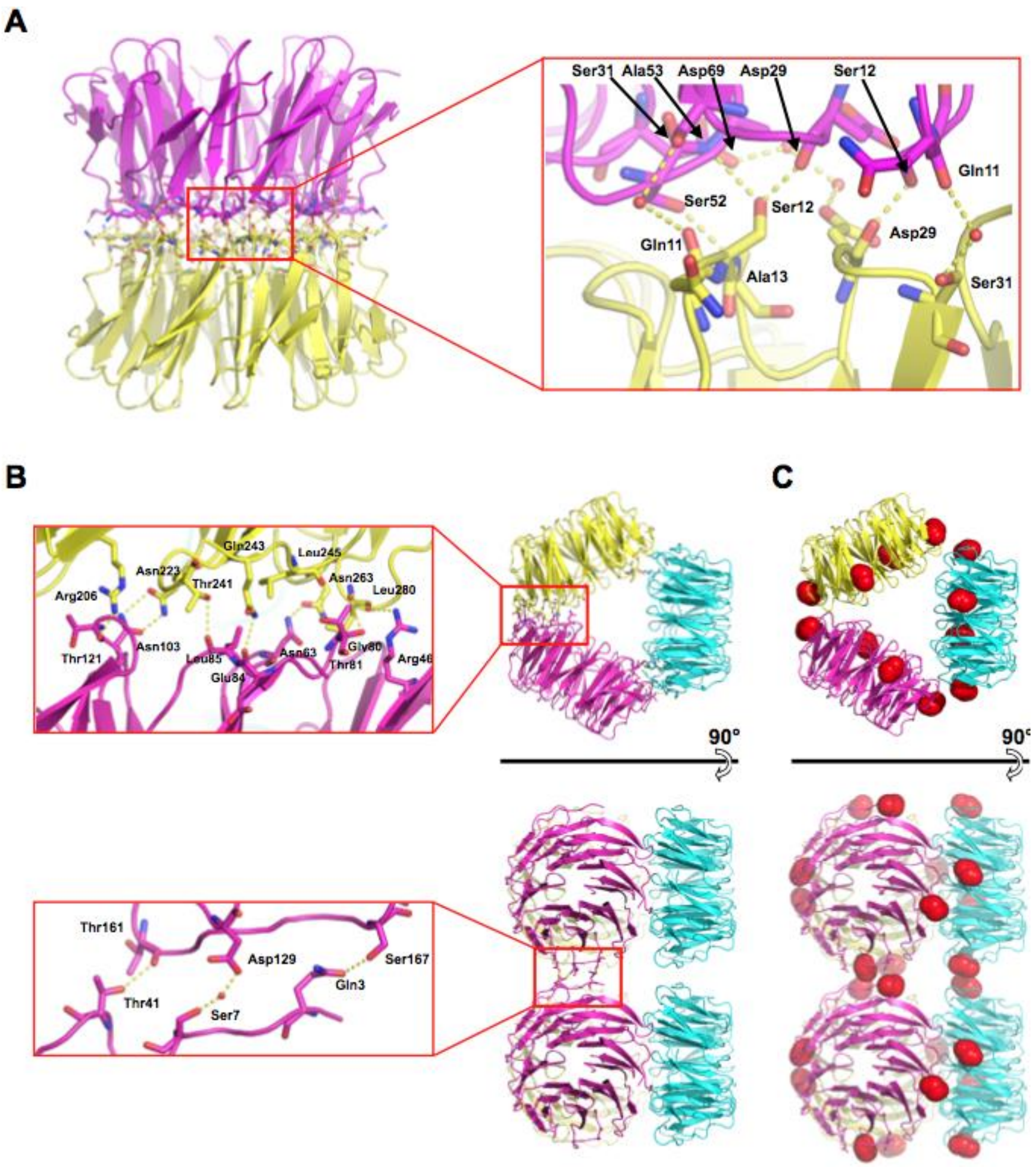

Figure S2 Conserved interaction mediating the crystal packing of the Tako and Ika proteins. In both crystal forms of the Tako8 protein a dimer can be observed (A). The interactions at the dimer interface are formed between a complex network of both direct or water-bridged hydrogen bonds, between every repeat of the opposing Tako8 proteins. The different orientations originate from different non-conserved water-mediated interactions at the outer rim of the dimeric Tako8 units. With the Ika proteins, a triangle formed by 3 Ika proteins can be observed (B). The interactions are formed by direct hydrogen bonding of the residues at the outer rim of the propeller unit. The triangles are stacked into pillars and stabilized by direct hydrogen bonds. These conserved interactions (both to form the trimer and the pillar stacking) are found close to the N/C termini (red spheres) of the theoretical Ika2 assembly and, this probably explains the failure of Ika2 to crystallise (C).

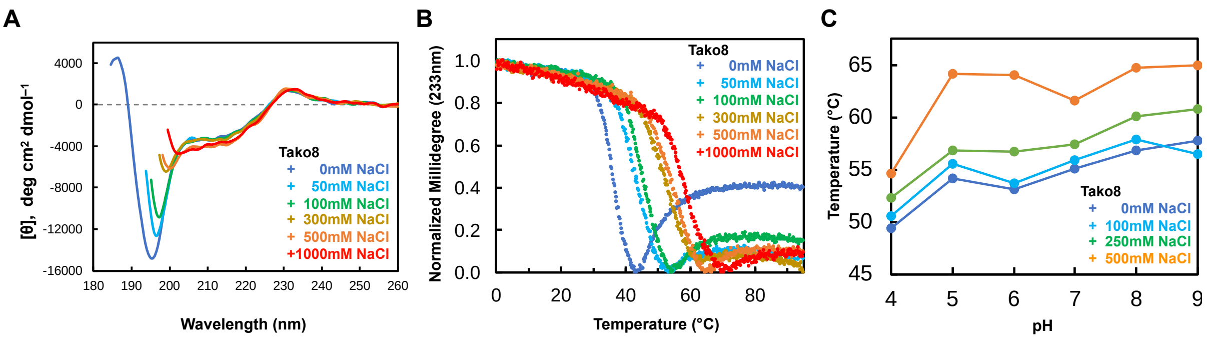

Figure S3 Influence of sodium chloride and pH on the stability of Tako8. In the presence of 1 M sodium chloride, Tako8 shows a CD spectrum similar to that of other well-folded β-propeller proteins such as Ika8. Reducing the salt concentration stepwise reveals a gradual loss of secondary structure (A). The melting temperature of Tako8 measured with different levels of salt present also shows a steady increase in thermal stability with salt concentration (B). To study the influence of pH on the Tako protein Differential Scanning Fluorimetry was used to measure the tertiary structure melting temperature. While at lower pH the protein stability is reduced, increasing salt concentrations lead to increased stability over the investigated pH range (C).

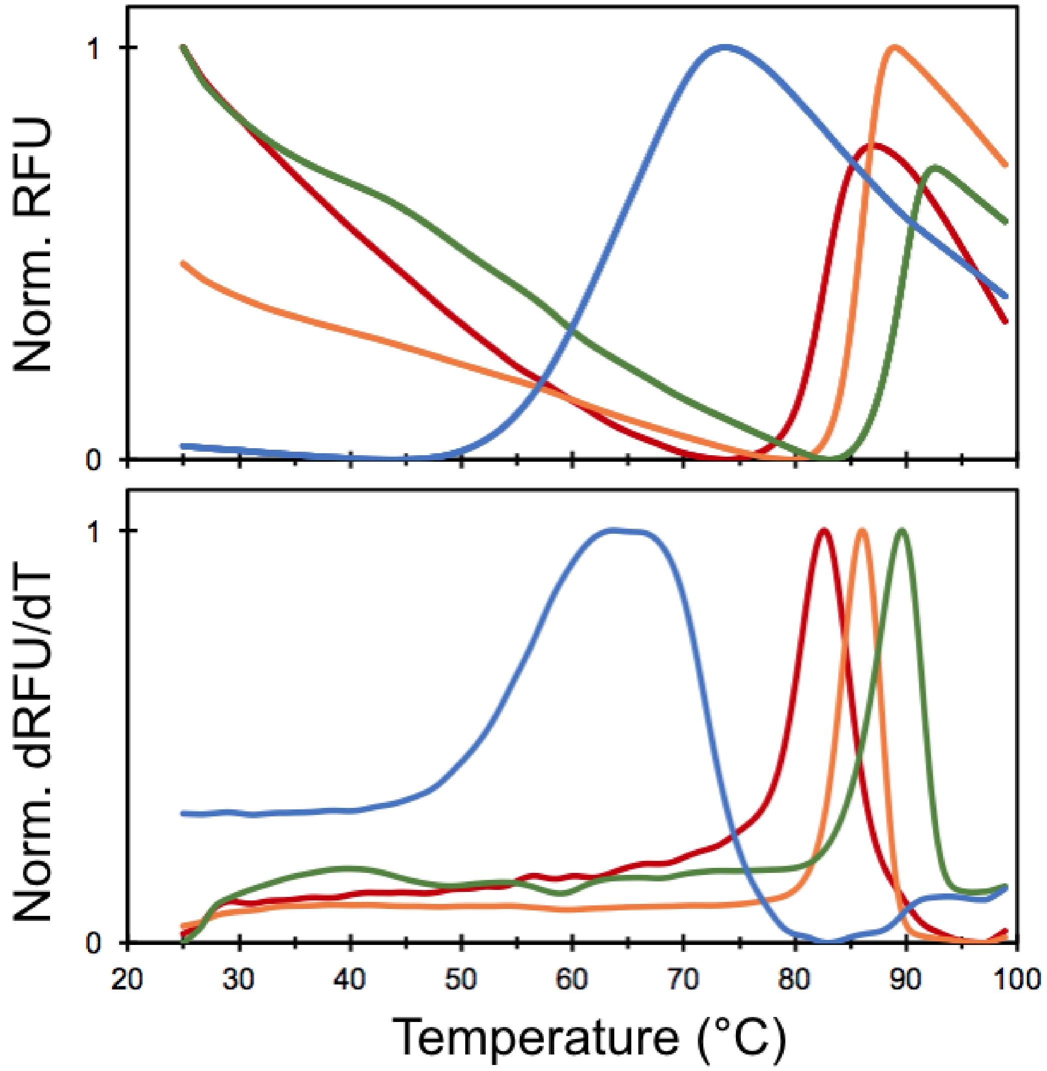

Figure S4 The differential scanning fluorimetry results of Tako and Ika proteins (Tako8-blue, Ika8-green, Ika4-orange and Ika2-red) dissolved in 20mM phosphate buffer pH7.6 (no NaCl). These results show similar trends to the thermal melting value measurement by CD (Fig. 2E). The lower diagram shows the derivative curve of raw data (above) and the peak indicates T_m value.

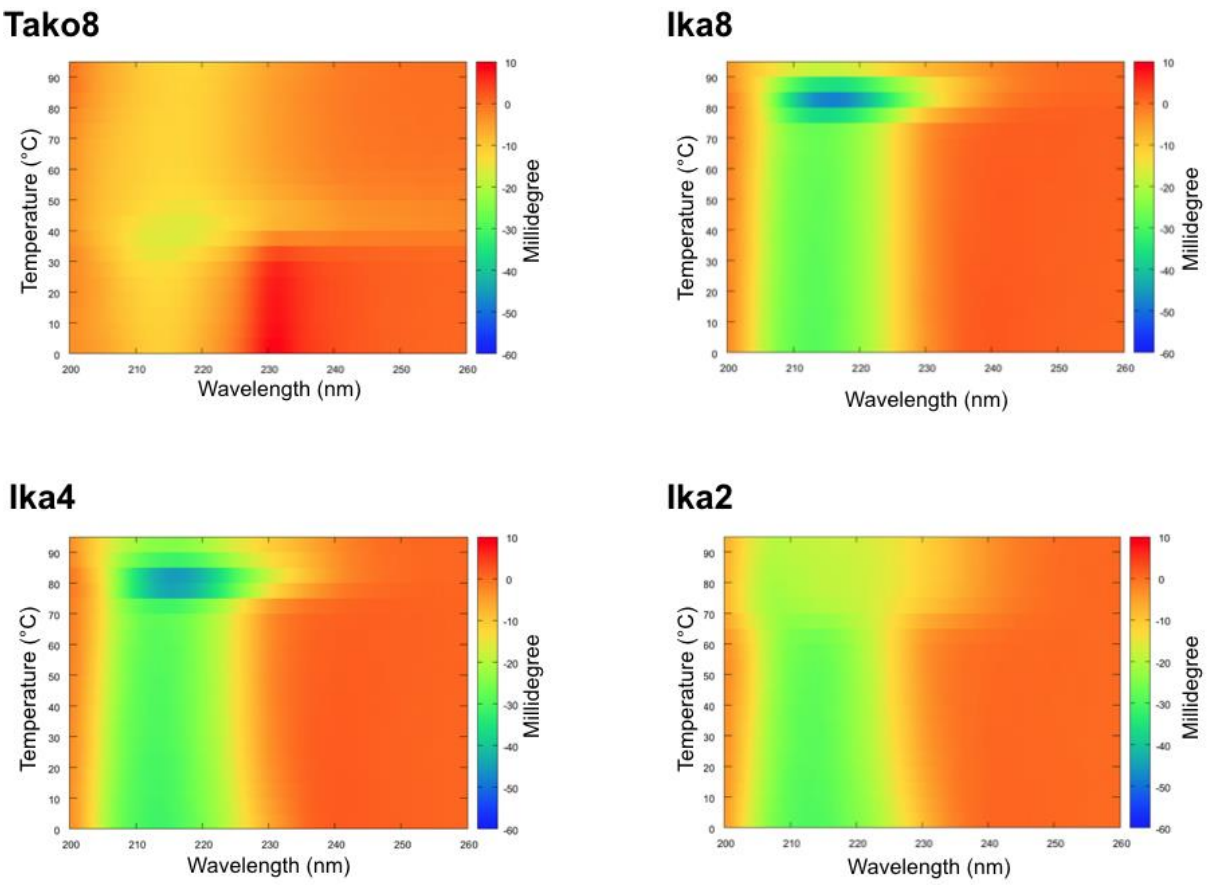

Figure S5 The CD spectrum change of Tako/Ika proteins by heating measured in 20mM phosphate buffer pH7.6 solution (no NaCl). The intensity of the CD signal is shown by colour change. The melting temperatures for Tako8, Ika8, Ika4 are approximately 40°C, 85°C and 80°C respectively. At higher concentration the Tako8, Ika8 and Ika4 proteins produce aggregates with a high content of β-strands.

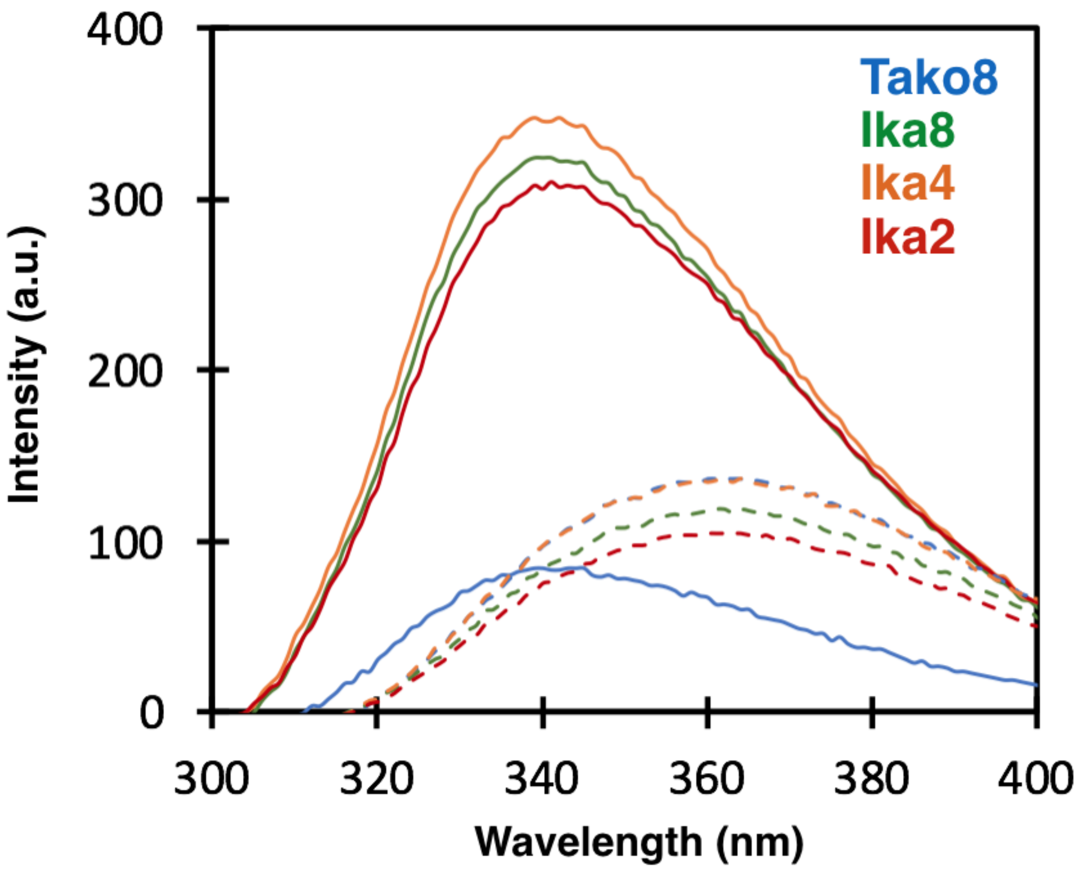

Figure S6 The intrinsic tryptophan fluorescence spectra (excited at 280 nm) before (solid line) measured in 20 mM phosphate buffer pH 7.6 solution (no NaCl) and after (dashed line) denaturation by 6 M GdnHCl. Denaturation of the proteins exposes the buried tryptophans to the solvent, which leads to a redshift in fluorescence. This behaviour makes the Tako and Ika proteins convenient models to study the folding of WD40 proteins.

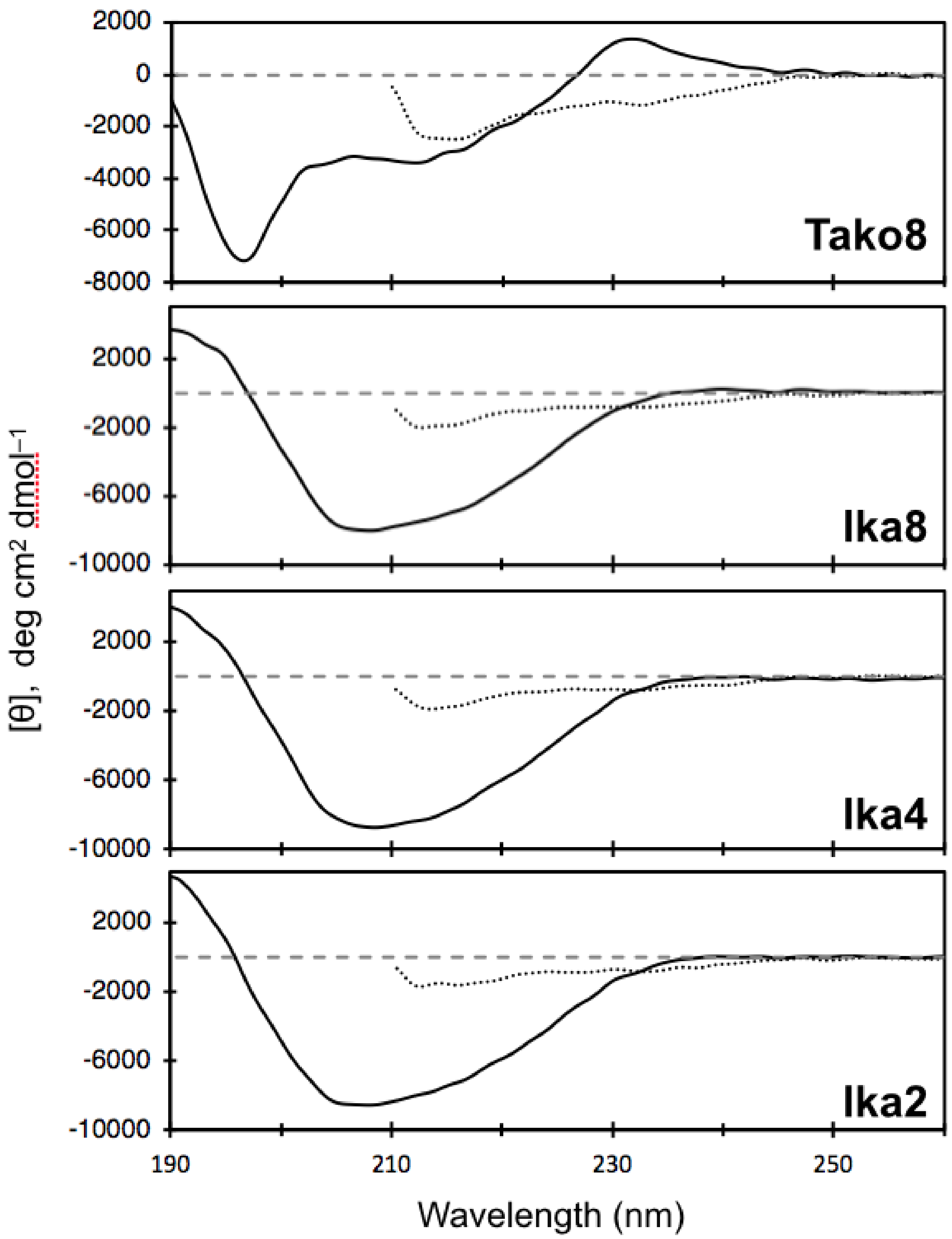

Figure S7 The CD spectra of folded Tako8, Ika8, Ika4 and Ika2 (solid line) in 20 mM phosphate buffer pH 7.6 solution (no NaCl) and after denaturation in 6 M GdnHCl solution (dashed line).
